# Fabrication and characterization of a novel corn straw/modified ammonium lignosulfonate bio-composite strengthened by polyethylenimine pretreatment

**DOI:** 10.1039/c9ra06237h

**Published:** 2019-10-29

**Authors:** Sidan Li, Yuan Yuan, Jinman Wang, Minghui Guo

**Affiliations:** Key Lab of Bio-based Material Science and Technology of Ministry of Education, Material Science and Engineering College, Northeast Forestry University Harbin 150040 China gmh1964@126.com; Heilongjiang Bayi Agricultural University Daqing 163319 China; Heilongjiang Provincial Key Laboratory of Environmental Microbiology and Recycling of Argo-Waste in Cold Region, College of Life Science and Technology, Heilongjiang Bayi Agricultural University Daqing 163319 China

## Abstract

This study focuses on the development of novel bio-composites *via* the pretreatment of corn straw particles (PCSP) and modified ammonium lignosulfonate (MAL) as a binder. The corn stalk particles (CSP) were pretreated with polyethylenimine (PEI) to enhance compatibility. The effects of PEI dosage on the mechanical properties and dimensional stability were examined, where PEI pretreatment improved the interfacial properties of MAL and CSP considerably. The optimum values of the PCSP/MAL composites complied with the Chinese national standard for load-bearing particleboard. Furthermore, the results confirmed that PEI pretreatment resulted in good surface activity and exhibited a favorable effect on the crystallinity of the PCSP/MAL composites. The storage moduli *E*′ and tan *δ* peak of the PCSP/MAL composites were considerably greater than those of the CSP/MAL composites. The fractured morphology of the composites clearly showed that PEI pretreatment improves the interfacial adhesion of MAL and CSP.

## Abstract 2

This study focuses on the development of novel bio-composites *via* the pretreatment of corn straw particles (PCSP) and modified ammonium lignosulfonate (MAL) as a binder.

## Graphical abstract

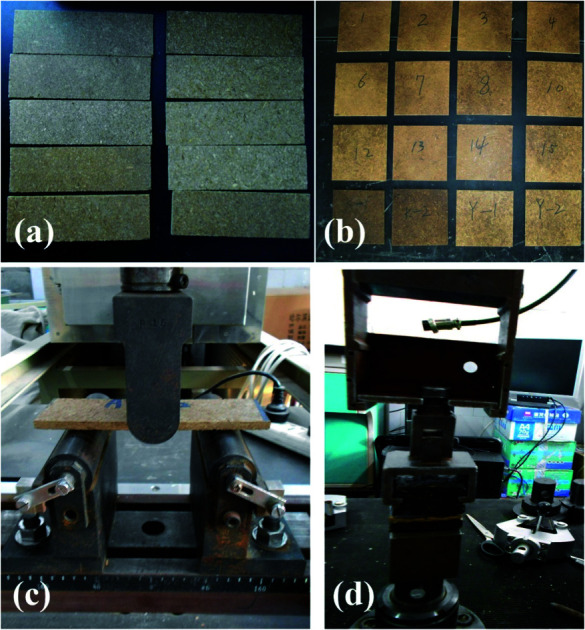

## 1 Introduction

The overwhelming depletion of our natural forests has spurred governments into proclaiming limits on the exploitation of wood sources. Hence, it has become increasingly important to manufacture board from non-wood sources. Different types of bindless boards from non-wood plant fiber sources have been developed, which include wheat straw,^1^ corn stalk,^2,3^ kenaf straw^4–6^ and rice straw.^7^ Zhou *et al.*^8^ developed an environmentally friendly thermal insulation material from cotton stalk fibers eliminating the need for resins and chemical additives. However, binderless boards have poor bond properties, mildew resistance and water resistance.^9^ These deficiencies limit their industrial applications,^10^ hence, various types of adhesives are employed in non-wood plant boards, such as UF resin,^11,12^ PF resin,^13^ MUF thermoset resin,^14^ and polymeric diphenylmethane diisocyanate (pMDI) resin.^9^

In accordance with the environmental protection act of China, formaldehyde emissions must be considerably reduced to improve existing board methods in 2015.^9^ However, few reports have depicted the development of formaldehyde-free panels, such as MDI. Importantly, although an alternative to formaldehyde, MDI exposure causes pulmonary diseases. A safe alternative adhesive is lignin adhesive,^15–18^ which is a new green formaldehyde-free adhesive^19–21^ that can be employed in non-wood commercial production. Lignin oxidation with H_2_O_2_ may effectively improve the solvent safe utilization because water is used to decomposition, which eliminates the need for organic solvents and reducing environmental issues. Furthermore, alkaline aqueous solutions are excellent reaction media compared to acidic or neutral environments.^22,23^ Corn straw are industrial raw material sources that have numerous potential applications, including energy, materials,^24^ and chemical production.^25^ The usage of agricultural residues originating from the forest industry can minimize air pollution caused by the combustion of corn residues, and increase the sustainability, as well as effectively reduce costs in the artificial panel manufacturing industry.^26^ However, most known residues, such as stalks and husks, are burned or disposed owing to multiple limitations, including farming conditions and collection cost.^14^

This paper investigates the feasibility of novel bio-composites *via* pretreatment of corn straw particles (PCSP) with a modified ammonium lignosulfonate (MAL) binder. Due to differences in polarity, corn straw requires pretreatment, but in order the make the procedure viable inexpensive reagents must be employed. In order to blend corn straw particles (CSP) and MAL homogeneously, polyethylenimine (PEI) is used efficiently to pretreat corn straw particles making them more compatible. However, little is known on whether PEI may improve the properties of CSP/MAL biocomposite. The purpose of this study is to demonstrate the effect of PEI pretreatment on characterization of the PCSP/MAL composites.

## 2 Experimental

### 2.1 Materials

Corn straw particles (moisture content: 5%) were obtained from Anda (Heilongjiang Province, China) and passed through a 40–60 mesh sieve for separation. The average chemical compositions of the initial particles were determined as 4.6% ash, 14.9% extractives, 16.7% lignin, 45.6% cellulose, and 22.5% hemicelluloses.^10^ Ammonium lignosulfonate (AL) was obtained from Shenyang Xingzhenghe Chemical Company (Shenyang, China) with the composition content determined as 51.9% total lignin, 27.1% carbohydrates, 10.6% ash, and 4.6% moisture. Polyethylenimine (PEI) was obtained from Shanghai UN Chemical (Shanghai, China). The molecular weight of PEI was 75 000 in 50 wt% aqueous solution. All other chemicals were of analytical grade.

### 2.2 Pretreatment of the corn straw particles (PCSP)

1 kg CSP of 40–60 mesh was placed into a closed blender, and 40 g PEI (2 wt% PEI) and 10 g glutaraldehyde were mixed homogenously and sprayed into the blender. Then the mixture was stirred at 50 °C for 30 min. They were then dried at 60 °C for 12 h to stop the pretreatment process. The target moisture content of PCSP was set between 4% and 6%.

### 2.3 Preparation of modified ammonium lignosulfonate (MAL)

MAL was prepared following a reported procedure:^23^ 1 g AL was dissolved in 10 mL distilled water and alkalinized adjusted to pH 10. Then, 10 mL H_2_O_2_ was mixed with the AL solution, which was stirred at 60 °C for 30 min. Thereafter, the mixture solution was concentrated to 20 wt% MAL solution.

### 2.4 PCSP/MAL board preparation

**Fig. 1:**
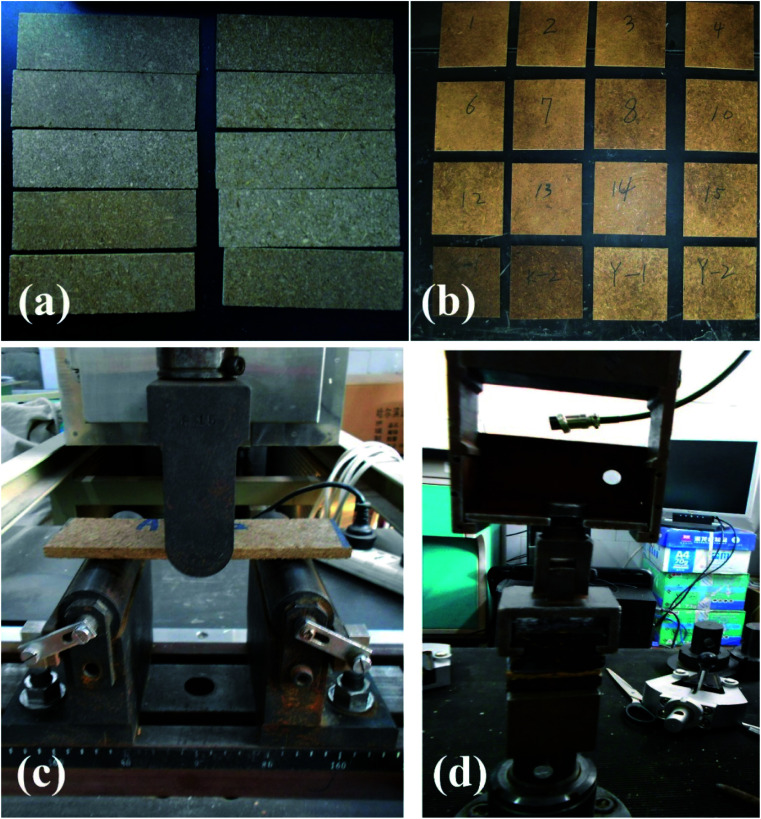
Photographs of the fabricated bio-composites: (a) samples of MOR and MOE with the dimensions of 200 mm × 50 mm × 5 mm, (b) samples of IB with the dimensions of 50 mm × 50 mm × 5 mm; (c) test mode of MOR and MOE; (d) test mode for IB.

The mass ratio of PCSP to MAL was 85 : 15 according to previous reports.^10^ The blender particles were hand-filled into the mat of a 300 mm × 300 mm forming box. The target density of each composite was determined as 0.8 ± 0.03 g cm^−3^ with a target thickness of 5 mm. Then the panels were stored under constant humidity at room temperature for 48 h. Fig. 1a and b show photographs of the prepared samples.

### 2.5 Mechanical and dimensional properties

The mechanical and dimensional properties of the composites were measured according to the Chinese National Standard GB/T 17657 (ref. 27) after conditioning at 20 ± 2 °C and 65 ± 5% relative humidity (RH). According to the standard, the ratio of bending moment and modulus under the maximum load is defined as modulus of rupture (MOR). The ratio of stress and strain under the load in the elastic limit is defined as modulus of elasticity (MOE). The internal bonding strength (IB) is the ratio of the maximum damage tension perpendicular to the sample surface of the sample surface area. The sample (200 mm × 50 mm) was performed by three-point static bending to measure the MOR and MOE values at a loading speed of 5 mm min^−1^. The sample (50 mm × 50 mm) was pulled apart in the vertical direction to measure the IB value at a loading speed of 2 mm min^−1^. Fig. 1c and d show the test methods for the mechanical properties of the composites. Three samples of each target composite were measured for reproducibility.

The thickness swell (TS) and water absorption (WA) were measured by the percentage increase in thickness and weight of the sample after 24 h of immersion in water at room temperature. 8 specimens (50 mm × 50 mm) of each target composite were analyzed. The surface hydrophobicity for each composite was measured at room temperature using JC2000A contact angle (CA). The load-bearing particleboard properties of GB/T 4897 (ref. 28) were determined as MOR ≥ 15 MPa, MOE ≥ 2200 MPa, IB ≥ 0.45 MPa, and 24 h TS ≤ 22%.

### 2.6 X-ray diffraction analysis (XRD)

The crystal structure and orientation of CSP and the prepared composites were studied using XRD with a wide angle. The 2*θ* angle ranges from 10° to 45° and set to 5° min^−1^ for the reflection mode scanning. The crystallinity index of the sample (15 mm × 15 mm × 3.2 mm) was calculated in accordance with previous reports.^29^

### 2.7 Dynamic mechanical analysis (DMA)

The storage modulus (*E*′) and tan *δ* curves of the CSP/MAL and PCSP/MAL composites were analyzed utilizing a thermal mechanical instrument (DMA-242 model). The three-point bending mold was performed for the test. The samples (50 mm × 8 mm × 5 mm) were heated from 50 to 250 °C at a rate of 5 °C min^−1^.

### 2.8 Scanning electron microscope (SEM)

The micrographs of the CSP/MAL and PCSP/MAL composites were obtained using Sirion 200 (FEI, the Netherlands). Each sample was coated with a thin layer of gold, and electrically conductive by an ion sputtering coater. SEM images were performed at 12.5 kV beam voltage.

## 3 Results and discussion

### 3.1 Effect of PEI dosage on the mechanical properties

**Fig. 2:**
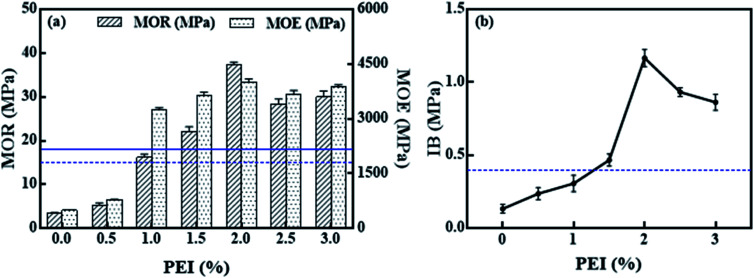
Effect of PEI treatment on the mechanical properties of corn straw bio-composites: (a) MOR and MOE, (b) IB.

The effect of PEI dosage on the mechanical properties is shown in Fig. 2. The straight line parallel to the *x*-axis manifests the minimum requirements of Chinese national standard.^28^

The effects of PEI dosage on MOR and MOE of the CSP/MAL composites are shown in Fig. 2a. MOR increases significantly with increasing binder content from 0 to 2 wt%. In the case of 2 to 2.5 wt%, a slight decrease in MOR occurs. However, increasing dosage from 2.5 to 3.0 wt%, gave no significant change in MOR. With increasing PEI dosage from 0 to 2.0 wt%, an intense increase in MOE is observed, whereas PEI dosage from 2.0 to 3.0 wt% did not display any such changes in MOE. When PEI dosage is greater than 1.0 wt%, MOR and MOE values exceed the minimum requirements (horizontal dashed and solid lines). Fig. 2b shows the effect of PEI dosage on IB of the CSP/MAL composites. There are two stages in the IB trend. Firstly, PEI dosage is in the range of 0–2 wt%, but with increases with increasing IB value. It is indicative of CSP crosslinking with PEI to improve bonding strength. In second stage of 2 to 2.5 wt%, a dramatic decrease in IB occurs. These results suggested that a high amount of PEI could lead to deterioration of mechanical performance of PCSP/MAL composites.

When PEI dosage is greater than 1.5 wt%, the IB values exceed minimum requirement (horizontal dashed line). By comparing 0% PF with 2% PF, the IB value increased eight fold, escalating from below 0.1 MPa to more than 0.4 MPa. After PEI pretreatment, MOR and MOE show an overwhelming increase. These significant changes in mechanical properties, especially IB, indicate that the interphase between MAL and CSP has been improved. The best values were obtained for 2% PEI: an MOR of 32.29 MPa, an MOE of 4001.15 MPa, and an IB of 1.17 MPa were measured, corresponding to an increase of approx. 996.76%, 712.14% and 775.00%, respectively, compared to the CSP/MAL composites without PEI pretreatment. Moreover, according to the above results, it's feasible to use MAL as adhesive instead of formaldehyde resin.

### 3.2 Effect of PEI dosage on the dimensional properties

**Fig. 3:**
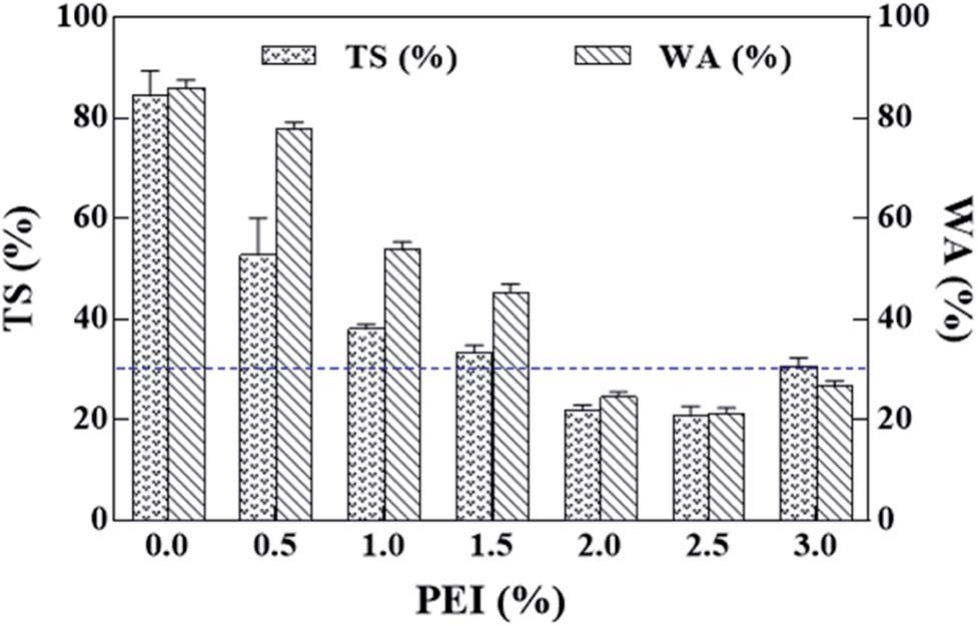
Effect of PEI treatment on the dimensional stability of corn straw bio-composites.

The effects of PEI dosage on the TS and WA values of the composites are shown in Fig. 3. In general, PEI-treated CS bio-board exhibits low TS and WA. When PEI dosage increases from 1.0 to 2.5 wt%, TS value decreases significantly, however, from 2.5–3.0 wt% TS value shows a remarkable increase. Improvements in the dimensional properties of biocomposites could be attributed to the hydrophobicity substances from MAL reacting with PEI of CSP surface, which prevented water from entering into its molecular chains.^23^ The TS must be lower than 22% for load-bearing particleboard.^28^ Furthermore, at 2.0 wt% and 2.5 wt% PEI dosage, the TS values meet minimum requirement (horizontal dashed line).

**Fig. 4:**
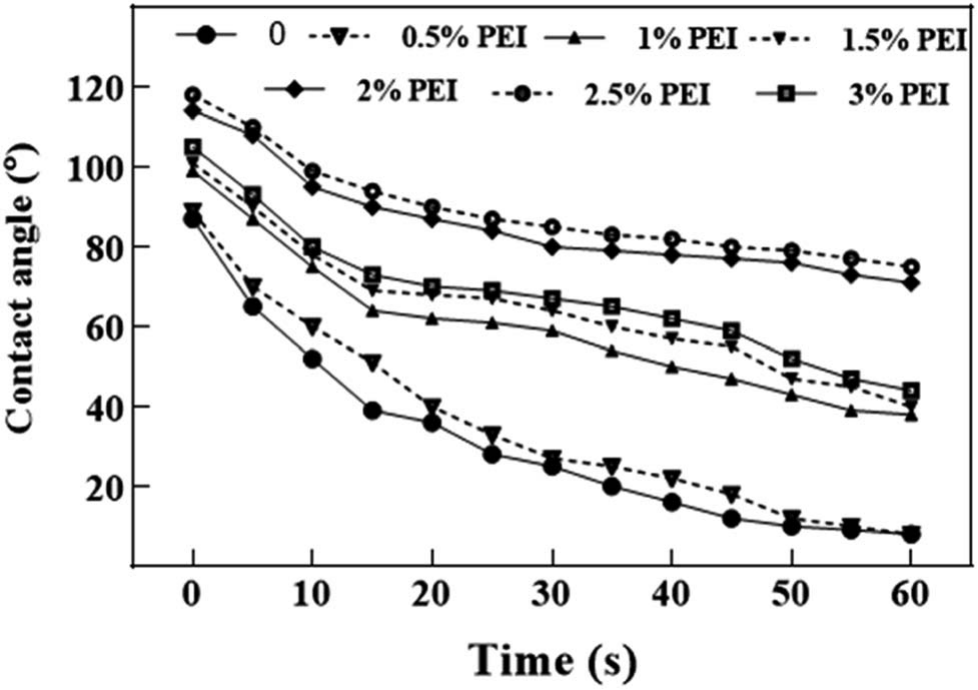
Effect of PEI dosage on CA at the surface of the composites.

The effect of PEI dosage on CA of the CSP bio-board with 15 wt% MAL is shown in Fig. 4. With increasing PEI dosage, the CA trend displays three distinct stages. Firstly, when PEI dosage is from 0 to 0.5 wt%, CA is approx. 90° to 25° after 30 s. The final CA is measured at approx. 8° after 60 s, which means very poor water resistance. Secondly, PEI dosage ranging between 1 and 1.5 wt% and 3 wt%, shows a change from 100° to 40° within 60 s, which also implies that the composite is hydrophilic. Furthermore, at dosage between 2 and 2.5 wt%, the CA is greater than 70° after 60 s, which indicates favourable hydrophobicity. Hence, the PEI dosage has a positive effect on the water resistance of the composites. This shows that poor interfacial adhesion makes the combination of MAL and CSP with too high or too low PEI not strong enough, and there maybe crakes, which leads to the infusion of water and subsequent fiber swelling. The suitable water resistance of the PCSP/MAL composites is 2–2.5 wt% of PEI dosage.

Before curing, the amine groups of PEI was grafted onto many –OH groups of CSP with the cross-linking of glutaraldehyde, meaning a high compatibility. Therefore, PEI can adhere well to the CSP surface. After curing, PEI of the corn straw surface could react with quinones of MAL enhancing strength and achieve good water-resistant. The binder mechanism is similar to the quinone–tannin processes in nature,^30^ where various reactions between the amino group and the catechol group solidify and crosslink the marine adhesive protein with strong and very water-resistant property.^31^ Hence active groups in MAL, such as phenolic hydroxyl and aldehyde groups, react with PCSP to form bonds.^32^ Hence, PEI significantly improves the interface properties of MAL and CSP, as well as their mechanical properties and dimensional stability.

### 3.3 X-ray diffraction analysis

**Fig. 5:**
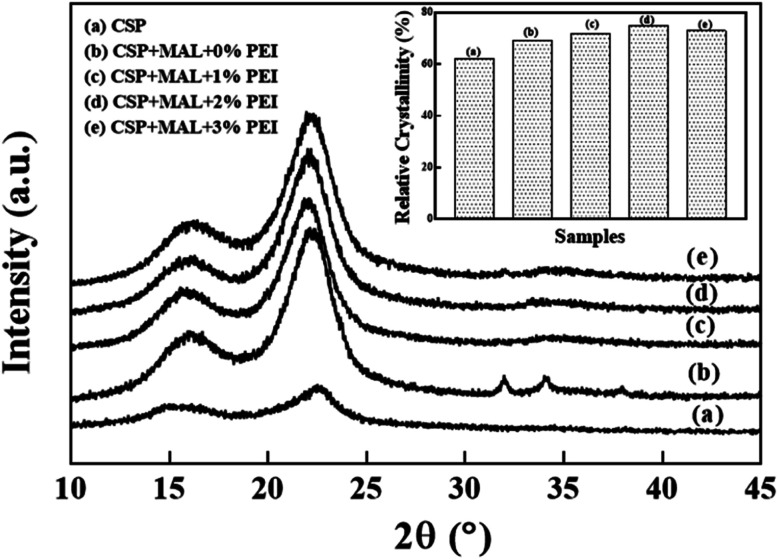
X-ray diffraction patterns obtained for the CSP, CSP/MAL and PCSP/MAL composites.

Fig. 5 shows the crystallinities of the CSP composites with different PEI content. Diffraction peaks of all samples are found at 2*θ* = 18.2° and 22.1° and are in accord with the typical cellulose I pattern.^33^ Therefore, the original crystal integrity of CSP cellulose is almost completely maintained during preparation. The diffraction peak of the sample is similar to that of natural cellulose, but differs from microwave-assisted,^34^ chemical^35^ and biological treatment,^36^ which changed the crystal integrity of cellulose.

The apparent crystallinity of the CSP is 61.96%, but after manufacturing with MAL, major alterations to the composites crystallinity occurs. In the absence of PEI pretreatment, the crystallinity of the CSP/MAL composite increases to 69.23%. At 1 wt% and 2 wt% PEI, the apparent crystallinity of the PCSP/MAL composite is further increased to 71.69% and 74.77%, respectively. This indicates that PEI possesses good surface activity while having a positive effect on the CSP composite crystallinity. Biopolymers such as cellulose and MAL contain a random array of crystalline and amorphous phases. However, introduction of PEI into the matrix may accelerate the nucleation step or lamellar rearrangement of the crystallization reaction of cellulose and MAL in the crystallization process, forming a small number of thinner crystals adhering to the original crystallites. Therefore, when PEI dosage is increased to 3 wt%, the crystallinity of CSP/MAL/PEI composite displays a slight decrease (72.87%). This shows that at higher PEI dosage adverse effects occur in crystallization of the PCSP/MAL composites.

### 3.4 Dynamic mechanical analysis (DMA)

**Fig. 6:**
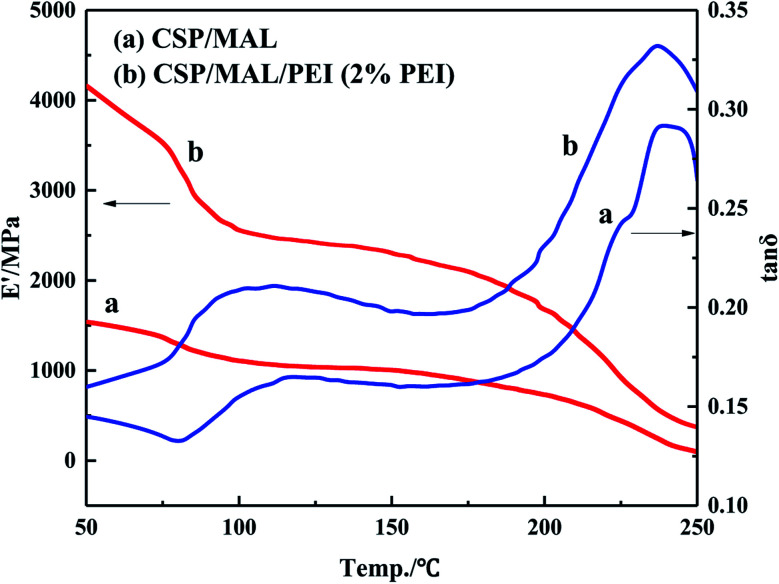
The storage modulus *E*′ and tan *δ* curves of CSP/MAL (a) and PCSP/MAL composites (b).

DMTA was employed to demonstrate the viscoelastic properties of the composites.^37,38^ The storage modulus *E*′ and tan *δ* curves of CSP/MAL and PCSP/MAL composites are shown in Fig. 6. *E*′ of PCSP/MAL composites is markedly higher than that of CSP/MAL composites. The maximum modulus of PCSP/MAL composites is 4159.76 MPa. The obtained results are consistent with the crystallinity data, as shown above in Fig. 5. The plots of tan *δ* value *versus* temperature are measured for the segmental motion of polymer molecules. Thus, the peak of tan *δ* is explained as Tg. The tan *δ* peaks for the composites are 237 °C. Hence, PEI pretreatment has no obvious influence on Tg. Moreover, the tan *δ* value of PCSP/MAL composites is 0.33 which appears higher than that of CSP/MAL composites, which suggests that PEI pretreatment influences the mobility of CSP chains during board manufacturing.^39^

### 3.5 Morphology of particles and composites

**Fig. 7:**
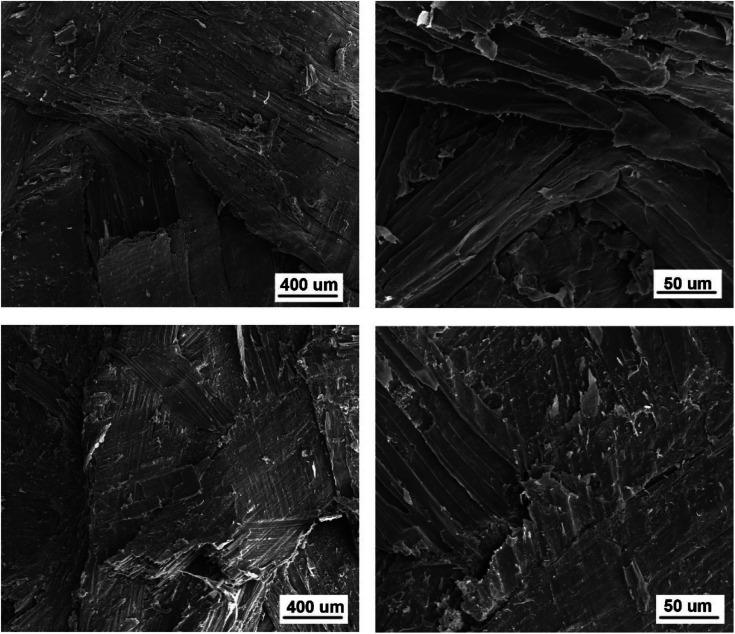
SEM images of the CSP/MAL (a and b) and PCSP/MAL composites (c and d).

Fig. 7 shows the micrographs of the CSP/MAL and PCSP/MAL composites at low and high magnifications, respectively. Without PEI pretreatment, the corn straw fiber cell walls almost retain their original morphology at high press temperatures.

Fig. 7a and b show that the CSP/MAL composites possess more voids (directed by the arrows). Due to CSP/MAL composites poor adhesion, the fibers are obviously debonded. Employing 2.0 wt% PEI pretreatment, the void ratio of PCSP/MAL composites is significantly reduced, which improves the compression properties of corn straw fibers, as shown in Fig. 7c and d. During the process of composite formation, the presence of PEI promotes the combination of the so-called glue line.^31^ Therefore, CSP treated by PEI strongly interacts with MAL molecules, this interaction is weaker when PEI treatment is not employed. This proves that PEI pretreatment improves the interfacial adhesion between MAL and CSP particles.

## 4 Conclusions

For the first time, new bio-composites were prepared using PCSP and MAL binders. In order to improve the interfacial adhesion between MAL and CSP, novel PEI treatment of CPS was adopted. The obtained results determined PEI pretreatment markedly improved the interfacial compatibility of the PCSP/MAL composites. Due to the rein-forcing effect of PEI surface treatment on the matrix, the PCSP/MAL composites showed an increase of 52.94–996.76% for MOR, 58.46–684.84% for MOE and 77.50–775.00% for IB compared with CSP/MAL composites (85/15). The optimum MOR, MOE, IB, TS and WA of the PCSP/MAL composites complied with the requirement of load-bearing particleboard, which were 37.29 MPa, 4001.15 MPa, 1.22 MPa, 21.77% and 24.52%, respectively. However, PEI negatively affected the mechanical properties and dimensional stability of the PCSP/MAL composites above 2.5 wt% PEI dosage. Furthermore, PEI displayed good surface activity and plays a positive role in improving the crystallinity of PCSP/MAL composites. This method may enable a new and low-cost method for the modification of straw particles and manufacturing of formaldehyde-free particleboards. These renewable and environmentally friendly composites may be suitable for commercial production. Further studies in this area will systematically focus on the bonding mechanisms of the interface.

## Conflicts of interest

There are no conflicts to declare.

## Supplementary Material
